# Oxidative Modification in the Salivary Glands of High Fat-Diet Induced Insulin Resistant Rats

**DOI:** 10.3389/fphys.2017.00020

**Published:** 2017-01-26

**Authors:** Urszula Kołodziej, Mateusz Maciejczyk, Agnieszka Miąsko, Jan Matczuk, Małgorzata Knaś, Piotr Żukowski, Małgorzata Żendzian-Piotrowska, Jan Borys, Anna Zalewska

**Affiliations:** ^1^Department of Conservative Dentistry, Medical University BialystokBialystok, Poland; ^2^Department of Physiology, Medical University of BialystokBialystok, Poland; ^3^Department of Histology and Embryology, Medical University of BialystokBialystok, Poland; ^4^County Veterinary InspectionBialystok, Poland; ^5^Department of Health Care Higher Vocational SchoolSuwalki, Poland; ^6^Department of Restorative Dentistry, Croydon University HospitalCroydon, England; ^7^Department of Hygiene, Epidemiology and Ergonomics, Medical University of BialystokBialystok, Poland; ^8^Department of Maxillofacial and Plastic Surgery, Medical University of BialystokBialystok, Poland

**Keywords:** insulin resistance, salivary glands, oxidative stress, oxidative damage, salivary dysfunction

## Abstract

Still little is known about the role of oxidative stress (OS) in the pathogenesis of the salivary gland dysfunction in the course of insulin resistance (IR). To induce IR rats was fed with a high fat diet (HFD) during 8 weeks. Stimulated and non-stimulated salivary flow rate, total protein, as well as oxidative damage markers: 4-HNE protein adduct, 8-isoprostanes (8-isoP), 8-hydroxy-D-guanosine (8-OHdG), advanced oxidation protein product (AOPP), and protein carbonyls (PC) were determined in the plasma and submandibular and parotid glands of IR and control rats. We have shown a significant decrease (45%) of the stimulated salivary flow rate, and in the total protein concentration in the parotid (35%) and submandibular (10%) glands of HFD IR as compared to the control rats. The level of 4-HNE protein adduct (15%) and 8-isoP (20%) in the submandibular glands of IR rats as well as total level of 4-HNE protein adduct (39%), 8-isoP (27%), AOPP (25%), PC (32%), and 8-OHdG (18%) in the parotid glands of IR rats were significantly higher as compared to the control group. We showed no correlation between the assessed OS parameters in the plasma and salivary glands. However, the redox balance in both glands shifted toward the oxidative status, parotid glands of IR rats are exposed to greater intensity OS. Stimulated secretory ability and mechanisms involved in the synthesis/secretion of proteins in the salivary glands are depressed in the course of IR. Oxidative damage in the salivary glands arises independently from the general OS in the course of insulin resistance induced by a high fat diet.

## Introduction

The fact that global population is becoming more obese due to the growing sedentary lifestyle and higher caloric intake results in an increase in insulin resistance (IR), which may be defined as reduced sensitivity to insulin in target tissues (Erejuwa, 2012). Due to the fact that insulin resistance is one of the earliest factor in the etiology of type 2 diabetes mellitus and often leads to the development of primary arterial hypertension and renal failure, it is considered an extremely serious health problem (Gan et al., 2013). Therefore, in recent years there has been a lot of interest, in this pathological states, both on the part of scientists, and clinicians.

One of the pathogenic factors leading to the development of IR is the phenomenon of oxidative stress (Aguirre et al., 2000; Kamata et al., 2005; Tinahones et al., 2009). According to Lushchak (2014), oxidative stress (OS) is “a situation where the steady-state reactive oxygen species (ROS) concentration is transiently or chronically enhanced, disturbing cellular metabolism, and its regulation and damaging cellular constituens.”

Excess production of ROS damages all important cellular components, such as DNA, lipids, and proteins, as well as disrupts cellular metabolism including altered gene expression, signal transduction, cell growth, and apoptosis. It has been shown that lipid peroxidation is the first, the most sensitive marker of oxidative damage which is related to the fact that lipids are very easily and immediately oxidized (Erel, 2005). The lipid peroxidation process changes the physical properties of the cell membranes—it reduces the hydrophobic interior, damages the spatial organization of the lipid membrane, as well as affects various cellular functions and pathways. Moreover, oxidation of lipids components leads to the lipid radical species that may damage other biomolecules. The result of the amino acids oxidation may be a cleavage of the polypeptide chain, as well as formation of crosslinks within one or more polypeptide chains (Stadtman and Levine, 2003). All of these result in a loss of activity and function of oxidatively modified proteins, with all biological consequences for the cell. It should be noted that ROS inhibit the thiol-containing (-SH) receptors, including muscarinic, adrenergic, serotonergic, and histaminergic receptors. Oxidative damage to DNA primarily affect the nitrogenous bases, sugar moieties, DNA strand breaks, as well as formation of the cross-links between DNA and proteins (Lovell et al., 2011). OS and its cytopathological consequences have been implicated in the onset and pathology of periodontitis (Pendyala et al., 2013; Öngöz Dede et al., 2016), oral precancer (Agha-Hosseini et al., 2012), and cancer (Bahar et al., 2007; Agha-Hosseini et al., 2012) as well as in the alteration of the salivary glands function in the course of general diseases (Su et al., 2010; Zalewska et al., 2014a, 2015; Knaś et al., 2016a,b).

They are many reports on the increased oxidative damage products in human or experimental animals plasma, liver, skeletal muscles, subcutanues, and viscelar adipose tissue in the course of the IR, type 2 diabetes and obesity (Keaney et al., 2003; Furukawa et al., 2004; Milagro et al., 2006; Grimsrud et al., 2007; Frohnert et al., 2011). Evidence implicates OS in the alteration of salivary glands function and composition in the course of type 2 diabetes (Al-Rawi, 2011; Aitken-Saavedra et al., 2015), or obesity (Narotzki et al., 2014; Giuseppe et al., 2015; Knaś et al., 2016b). However, there are hardly any studies analyzing OS in the salivary glands in the course of IR. Zalewska et al. (2014b) showed alterations in the antioxidant enzymatic system of the salivary glands of rats with high fat diet (HFD) induced insulin resistance but did not prove if OS is clearly present. It is commonly accepted that the most reliable evidence of induction of OS is increased concentrations of oxidatively changed biomolecules, however changes in the activity or concentrations of antioxidants are being challenged in this regard.

This study is aimed at examining the OS level in the salivary glands of HFD induced insulin resistance and control rats by assessing the concentration of essential markers of oxidative damage as well as association between oxidatively modified cellular components and the secretory ability in the parotid and submandibular glands of rats in the course of HFD induced insulin resistance.

## Materials and methods

The experimental procedures concerning animal treatment and maintenance were approved by the institutional Committee for Ethics use of Animals in the Medical University in Bialystok, Poland (protocol number 89/2015, 2015/109).

After arrival at the experimental animal house at the Department of Physiology, Medical University in Bialystok, Poland, the animals underwent 1-week adaptation period to the new conditions. Next, 16 male Wistar rats were divided randomly into two groups: control (C, *n* = 8) and experimental (HFD-IR, *n* = 8). The rats were housed in standard cages and maintained at controlled temperatures (20–21°C), under standard condition of light from 6.00 a.m. to 6.00 p.m. and with free access to tap water.

### Diet

During the adaptation period, all rats were fed a standard diet which comprised 10.3% fat, 24.2% protein, and 65.5% carbohydrates (kcal) (Agropol, Motycz Poland).

For eight consecutive weeks of the experiment, the control group received the same standard diet (Agropol, Motycz Poland) as in the adaptation period. To develop insulin resistance (Qu et al., 2008; Gan et al., 2013), the HFD-IR group was fed a HFD (Research Diets, INC cat no. D12492) which was composed of 59.8% fat, 20.1% protein, and 20.1% carbohydrates (kcal) and had unlimited access to water.

The rats were weighed for the first time on the day of their arrival at the animal house and immediately prior to sacrifice.

After overnight fasting, rats were anesthetized by intraperitoneal injection with phenobarbital (80 mg/kg of body weight). Animals were placed on the heated couch (37°C) in the supine position to evaluate the salivary secretory ability. Whole non-stimulated saliva was collected from the oral cavity with the pre-weighted cotton balls for 15 min by the one experienced person (Sabino-Silva et al., 2013). Whole stimulated saliva was collected under parasymphatetic stimulation. Animals were injected with pilocarpine nitrate (5 mg/kg BW, intraperitoneal, Sigma Chemical Co, St. Louis, MO, USA). Five minutes after the pilocarpine administration stimulated whole saliva was collected, for 5 min (Picco et al., 2012). The salivary secretory ability was determined from the difference in the initial and final weight of the cotton balls. We assumed that 1 mg is equal to 1 μL (Romero et al., 2012).

Subsequently, whole blood was collected from the abdominal aorta and the salivary glands were removed. The right salivary glands were weighed (laboratory weight KERN PLI 510-3 M), immediately freeze-clamped with aluminum tongs, frozen in liquid nitrogen and stored at −80°C until biochemical determinations. The left salivary glands were fixed with 10% formalin solution.

Blood was harvested into glass tubes with heparin and centrifuged (5 min, 4°C, 3000 g, MPW 351, MPW Med. Instruments, Warsaw, Poland). The obtained plasma was frozen in liquid nitrogen and stored at −80°C. There was no haemolysis in any of the obtained plasma.

Directly before the determinations, the salivary glands and plasma were thawed (4°C), cut into small pieces and diluted (1:10) in ice cold PBS [to assess concentrations of the carbonyl groups, part of the salivary glands were diluted in 50 mM phosphate buffer (1:10)]. Then, the salivary glands were homogenized with the addition of the protease inhibitor (1 tablet/10 mL of the buffer; Complete Mini Roche, France) and the addition of antioxidant butyl-hydroxytoluene (10 μL 0.5 M BHT in acetonitryle per 1 mL of the buffer; BHT; Sigma-Aldrich, Germany), on ice using glass homogenizer (Omni TH, Omni International, Kennesaw, GA, USA), and sonificated (1800 J/sample, 20 s three times, on ice; ultrasonic cell disrupter, UP 400S, Hielscher, Teltow, Germany). For plasma samples, solution of BHT and protease inhibitor were also added. The resulting homogenates were spinned for 20 min, 4°C, 5000 g (MPW Med Instruments, Warsaw, Poland). Only supernatants were further analyzed the same day.

### Assays

The plasma free fatty acids (FFA), insulin, glucose concentrations and plasma and salivary glands 4-HNE protein adduct, 8-isoprostanes (8-isoP), 8-hydroxy-D-guanosine (8-OHdG), advanced oxidation protein product (AOPP), protein carbonyls (PC), and total proteins concentrations were performed in duplicates. The final result is the arithmetic average of the two measurements. Results were converted to the grams of the total protein. Normalisation to total protein is used to observe the differences in the ratio of biochemical parameters present in the salivary glands or plasma.

FFA were determined by the method Bligh and Dyer (1959), the fasting glucose was analyzed by glucometer (Accu-check glucometer, Byer, Germany), the insulin level was assessed by the ELISA method (BioVendor, Brno, Czech Republic). Based on these results, the insulin sensitivity was calculated using the HOMA index of insulin resistance (HOMA-IR) = fasting insulin (U/mL)x fasting glucose (mM)/ 22.5 (Ebertz et al., 2014).

The lipids (8-isoP, 4-HNE protein adduct) and DNA (8-OHdG) oxidations products were determined using commercial ELISA kits (Cell Biolabs, Inc. San Diego, CA, USA; Cayman Chemicals, Ann Arbor, MI, USA; USCN Life Science, Wuhan, China, respectively) according to the manufacturer's instructions. The absorbance of the colored reaction product was measured at 405 nm using a microplate reader MINDRAY MR- 96A.

The PC was assessed as described previously (Reznick and Packer, 1994). Briefly the supernatant and plasma were incubated for 60 min, 25°C with 10 mM DNPH (2,4-dinitrophenylhydrazine; POCH. SA (Polskie Odczynniki Chemiczne. Spółka Akcyjna, Gliwice, Poland) dissolved in 2.5 M HCl. The concentration of PC was determined spectrophotometrically, in the presence of the blank (guanidine hydrochloride), by measuring the absorbance at 355 nm and using the molar absorption coefficient for DNPH ε = 22,000 M^−^1 cm^−1^.

AOPP were determined colorimetrically according to the method (Kalousová et al., 2002). Two hundred microliter of tissue homogenate and serum diluted 1:5 in PBS were incubated with 10 μL of 1.16 M potassium iodide (Sigma-Aldrich, Germany) and 20 μL of glacial acetic acid (POCH SA, Gliwice, Poland). The absorbance of the mixture was measured immediately at 340 nm. A standard curve was made for chloramine T (Sigma-Aldrich, Germany) and the results were expressed in chloramine units per mg of total proteins.

The bicinchioninic method was used to determine the protein concentration. The bovine serum albumin was used as a standard (Thermo Scientific PIERCES BCA Protein Assay Kit, Rockford, IL, USA).

### Histological examination

The left salivary glands were embedded in paraffin for making five micron sections. Next, the sections were stained with hematoxylin-eosin and examined under light microscope (OPLYMPUS BX 51, OLYMPUS) using a gratitude 40 and 60 x magnification. Histological examination was carried out by a histologist.

### Statistical analysis

The data were expressed as median, minimum and maximum. Statistical analysis was performed using Statistica 10.0 (Statsoft, Cracow, Poland). For statistical analyses, the groups were compared using the nonparametric test (U Mann-Whitney test). The Spearman Correlation Coefficient was used to study the associations between the OS markers, protein concentrations in the salivary glands and blood glucose, insulin and FFA concentrations as well as salivary flow rate. The statistical significance was defined as *p* ≤ 0.05.

## Results

### Effects of high fat feeding on the body weight, plasma glucose, free fatty acids, and insulin concentration and salivary glands weight

Despite the fact that average daily food intake was similar in both groups, HFD-IR rats presented with a higher body weight when compared to the control rats (*p* = 0.0002) (Table 1). However, when comparing the HFD-IR and the control salivary glands' weight there was a significant increase only in the case of the parotid glands of HFD-IR group as compared to the control parotid glands (*p* = 0.041) (Table 1). The 8-week high fat intake also affected glucose homeostasis. We observed an increase in the fasting glucose concentration (*p* = 0.001) as well as in the insulin concentration (*p* = 0.0009) in the HFD-IR rats in comparison with the control rats. Moreover, the insulin sensitivity calculated by HOMA-IR, allowed us to conclude that high fat feeding results in insulin resistance. The median value of the HOMA-IR index was significantly higher in the group of HFD-IR rats as compared to the reference rats (*p* = 0.00001) (Table 1).

**Table 1:** **Effect of 8-weeks high fat feeding on body weight, fasting plasma glucose, insulin, and FFA level, HOMA-IR, salivary: glands weights, flow, and total proteins**.

	**Body weight (g) at the beginning/at the end of the study M (min-max)**	**Glycaemia (mg/dL) M (min-max)**	**Insulin (mU/mL) M (min-max)**	**FFA (μmol/L) M (min-max)**	**HOMA-IR M (min-max)**	**Salivary gland weight (mg) parotid/submandibular M (min-max)**	**Salivary flow rate (μL/min) unstimulated/stimulated M (min-max)**	**Total protein (mg/dL) parotid/submandibular M (min-max)**
C *n* = 8	50(46–57)/314.7(273–331)^*^	95.3 (91–101.3)^*^	1.14(0.79–1.35)^*^	83.4(62.3–94.1)^*^	4.87(3.2–7.6)^*^	0.087(0.076–0.09)^*^/0.25(0.23–0.285)	0.4(0.11–0.66)/118.49(86.88–193.3)^*^	5037.1(4188.2–7553.6)^*^/4819(4679–5251.1)^*^
HFD-IR *n* = 8	53(46–61)/407.7(376–450)	175.6(125.1–210.5)	2.69(2.19–2.83)	185.9 (157.5–198.4)	21.0(12.2–26.5)	0.108(0.093–0.11)/0.27(0.22–0.34)	0.39(0.07–0.59)/65.11(35.6–79.89)	3274.9(3108.4–3998.9)/4337(3606.2–4592.9)

C, control; HFD-IR, high fat diet insulin resistant rats; FFA, free fatty acids; HOMA-IR, HOMA index of insulin resistance;

* *p < 0.05; M (min-max)- Median (minimum-maximum)*.

Finally, rats fed with HFD were characterized by an elevated plasma free fatty acids concentration as compared to the control rats (*p* = 0.001).

### The effect of high fat feeding on plasma 4-HNE-protein adduct, 8-isoP, 8-OHdG, AOPP, and PC

**Figure 1:**
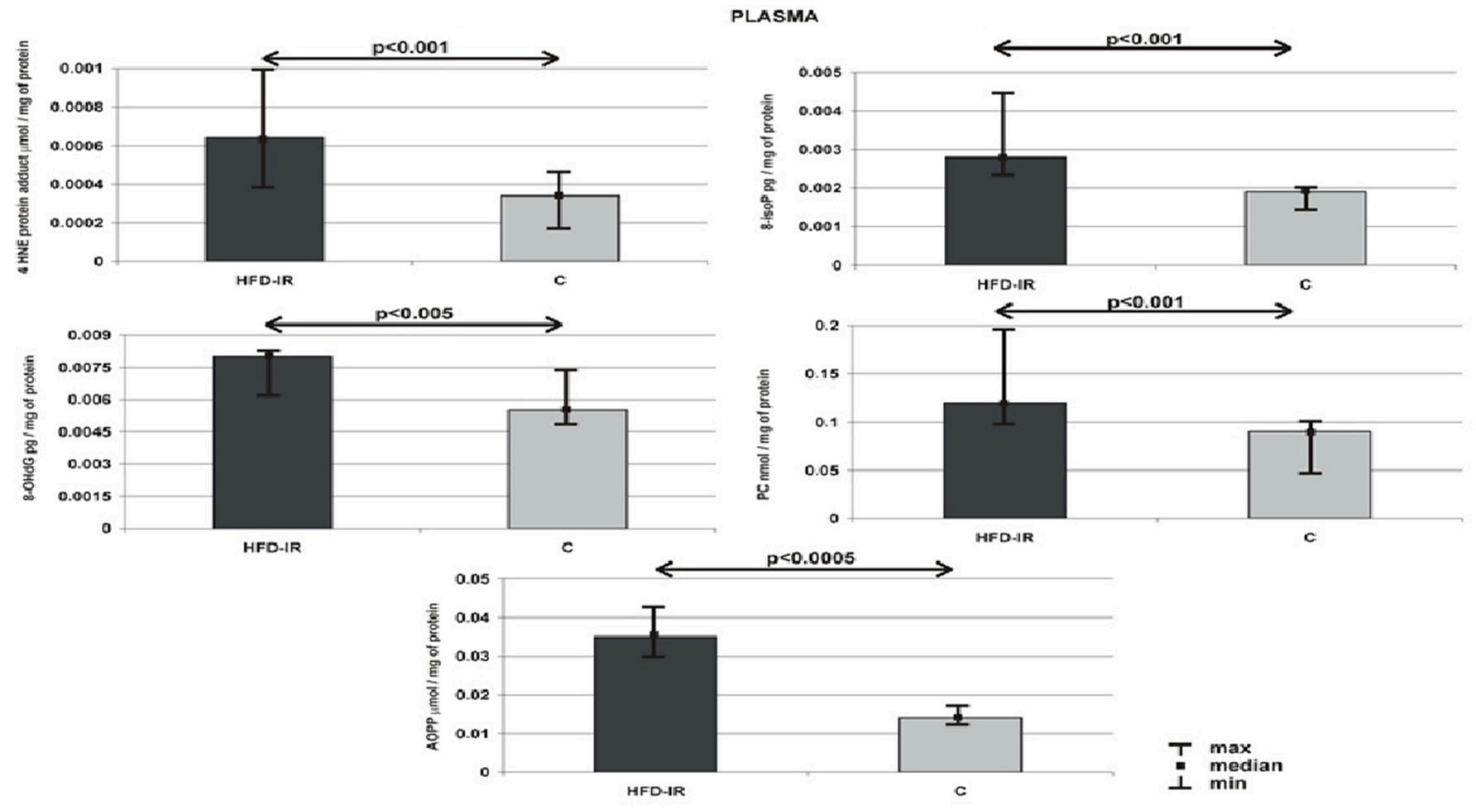
**Plasma 4-HNE protein adduct, 8-isoP, 8-OHdG, PC and AOPP of the HFD-IR and control rats**. M, median; min, minimum; max, maximum; HFD-IR, high fat diet- insulin resistant rats; C, control group; 8-isoP, 8-isoprostanes; 8-OHdG, 8-D-hydroxyguanosine; PC, protein carbonyl groups; AOPP, advanced oxidation protein products.

Plasma 4-HNE-protein adduct, 8-isoP, 8-OHdG, AOPP and PC concentration of HFD-IR and control rats are given in Figure 1. The obtained results proved that 8-week high fat feeding increased the general oxidative stress. We have shown that comparison of plasma OS biomarkers within groups revealed that HFD-IR group presented an elevated plasma 4-HNE-protein adduct (51%, *p* = 0.0008), 8-isoP (37%, *p* = 0.0007), 8-OHdG (27%, *p* = 0.003), AOPP (169%, *p* = 0.00043), and PC (42%, *p* = 0.0005) as compared to the control rats.

### The effect of high fat feeding on salivary flow rate and protein concentration in the parotid and submandibular glands

Our results showed that 8-week high fat feeding had only a minor effect on the unstimulated secretory ability of the salivary glands HFD-IR rats as compared to the control group, but significantly reduced the stimulated flow rate compared with the control group (*p* = 0.0003) (Table 1).

On the contrary, we observed that, high fat feeding influenced protein concentrations in both salivary glands. As shown in Table 1 protein concentration in the parotid (35%) and submandibular (10%) glands' tissue of HFD-IR rats decreased significantly when compared with that of the control glands (*p* = 0.031, *p* = 0.0009, respectively; Table 1).

### Submandibular glands

**Figure 2:**
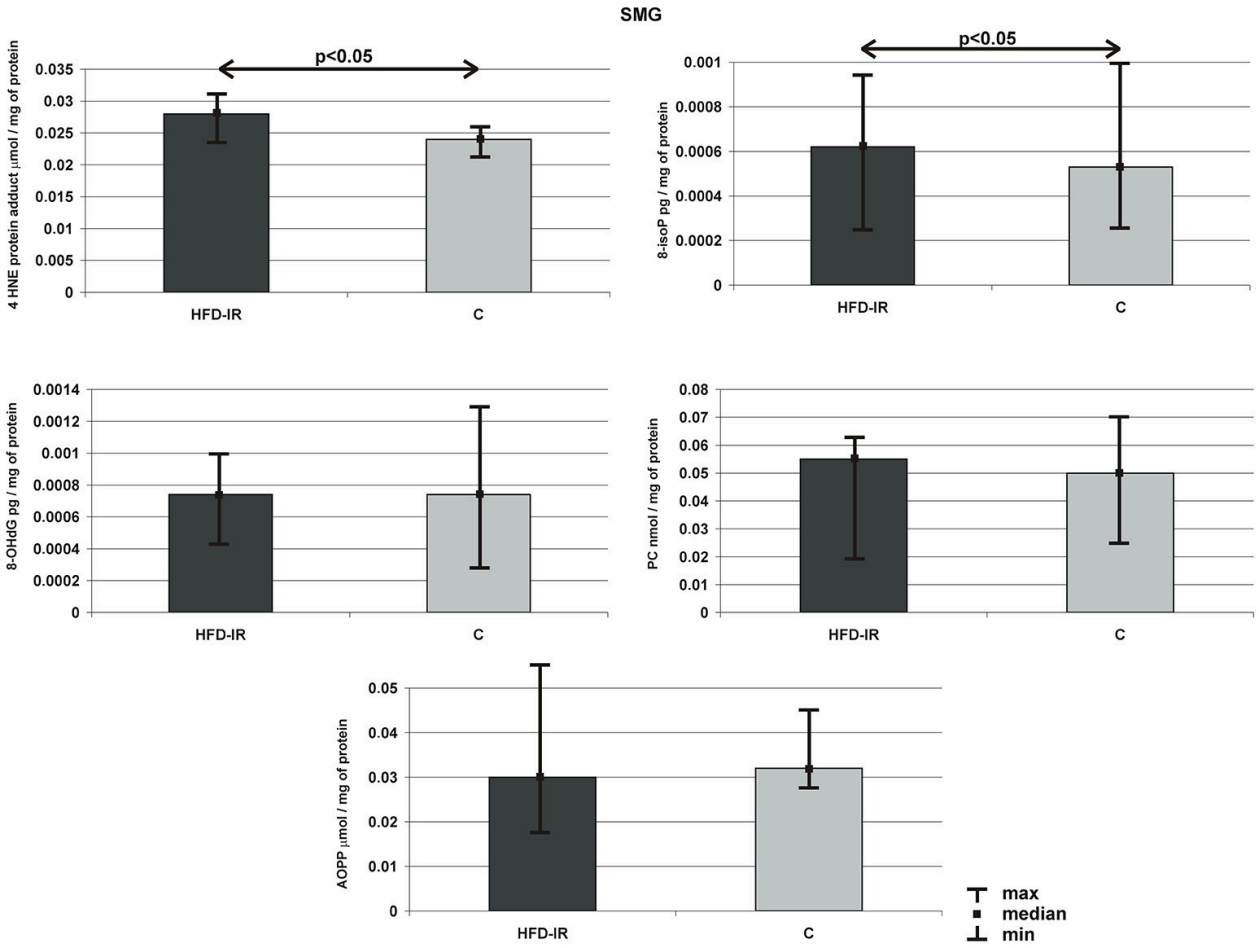
**Submandibular 4-HNE protein adduct, 8-isoP, 8-OHdG, PC, and AOPP of the HFD-IR and control rats**. SMG, submandibular glands; M, median; min, minimum; max, maximum; HFD-IR, high fat diet insulin resistant rats; C, control group; 8-isoP, 8-isoprostanes; 8-OHdG, 8-D-hydroxyguanosine; PC, protein carbonyl groups; AOPP, advanced oxidation protein products.

Submandibular glands 4-HNE-protein adduct, 8-isoP, 8-OHdG, AOPP and PC concentration of HFD-IR and control rats are presented on Figure 2. Submandibular glands: 4-HNE-protein adduct (15%) and 8-isoP (20%) concentrations of HFD-IR rats increased significantly as compared to the control rats (*p* = 0.02, *p* = 0.03, respectively). On the other hand, the submandibular glands of the HFD-IR and control rats were comparable in terms of 8-OHdG, AOPP and PC concentration.

### Parotid glands

**Figure 3:**
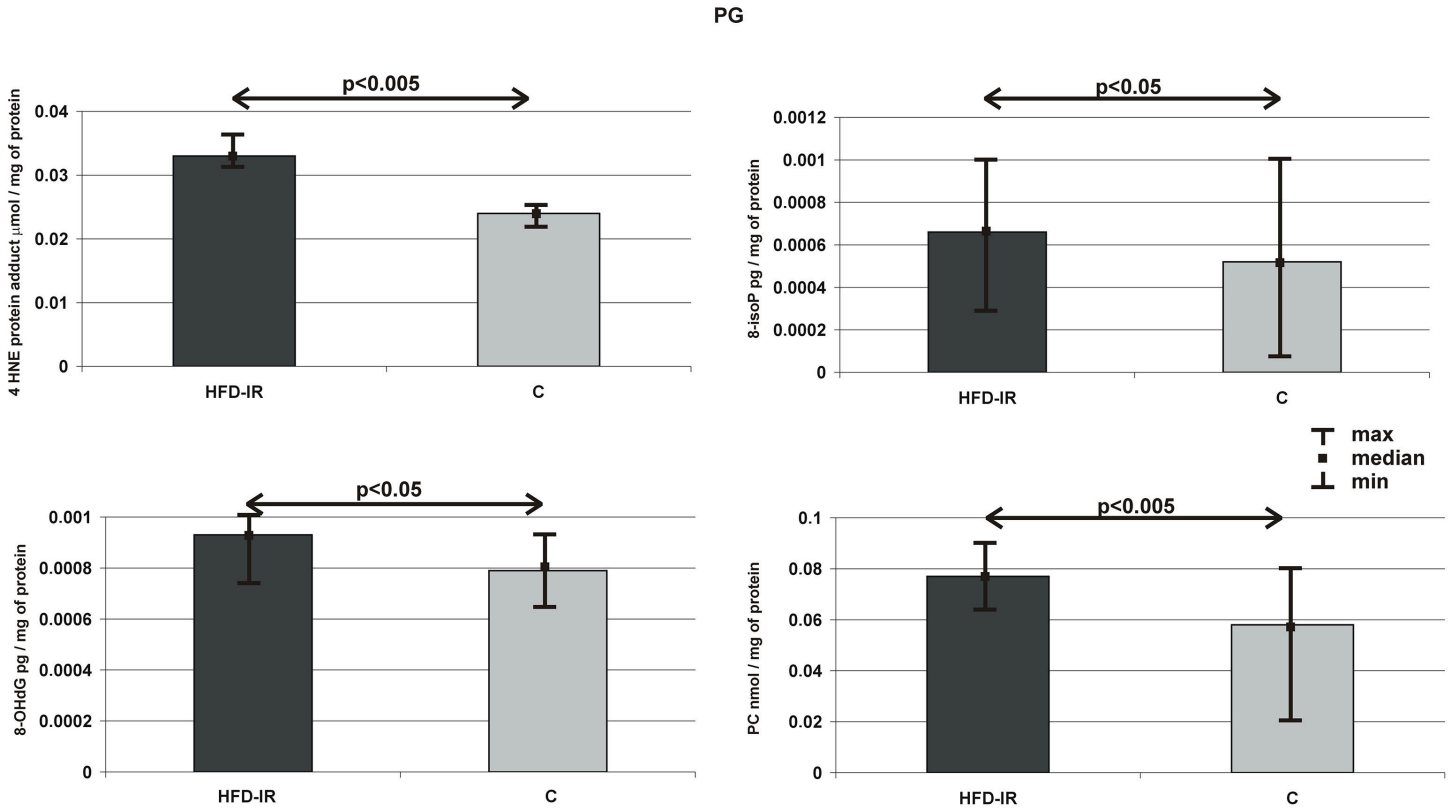
**Parotid 4-HNE protein adduct, 8-isoP, 8-OHdG, PC, and AOPP of the HFD-IR and control rats**. PG, parotid glands; M, median; min, minimum; max, maximum; HFD-IR, high fat diet insulin resistant rats; C, control group; 8-isoP, 8-isoprostanes; 8-OHdG, 8-D-hydroxyguanosine; PC, protein carbonyl groups; AOPP, advanced oxidation protein products.

Figure 3 summarizes the parotid gland markers of OS in the HFD-IR and control groups. On the contrary to submandibular glands, parotid glands of HFD-IR rats showed the severity of oxidative modifications of all types of cellular elements, which we observed in the form of significant increases in concentrations of 4-HNE-protein adduct (39%), 8-isoP (27%), 8-OHdG (18%), AOPP (25%), and PC (32%) when compared to the control group (*p* = 0.003, *p* = 0.03, *p* = 0.035, *p* = 0.023 *p* = 0.002, respectively).

### Parotid vs. submandibular

Comparison of parotid and submandibular glands OS biomarkers revealed quite different results within control and HFD-IR groups (Table 2). Our results showed that the control parotid and submandibular glands were comparable concerning all examined parameters of the oxidative stress. On the contrary, the cellular elements of the parotid glands of HFD-IR rats underwent more intensive oxidative modification as compared to the submandibular ones. The median concentrations of the parotid glands: 4-HNE-protein adduct, 8-isoP, 8-OHdG, AOPP and PC increased significantly as compared to the submandibular glands (*p* = 0.02, *p* = 0.04, *p* = 0.043, *p* = 0.03, *p* = 0.001, respectively).

**Table 2:** **Differences between submandibular and parotid salivary glands**.

**Group**		**SMG M (min-max)**	**PG M (min-max)**	**P SMG: PG**
HFD-IR	4-HNE-protein adduct (μg/mg of protein)	0.027 (0.024–0.031)	0.034 (0.037–0.029)	0.02
	8-isoP (pg/mg of protein)	0.00062 (0.00026–0.00094)	0.00066 (0.00031–0.001)	0.04
	8-OHdG (pg/mg of protein)	0.00074 (0.00042–0.00096)	0.00093 (0.00073–0.001)	0.03
	PC (nmol/mg of protein)	0.055 (0.019–0.062)	0.077 (0.065–0.091)	0.001
	AOPP (μmol/mg of protein)	0.03 (0.018–0.056)	0.035 (0.029–0.037)	0.03
C	4-HNE-protein adduct (μg/mg of protein)	0.023 (0.021–0.026)	0.024 (0.022–0.025)	ns
	8-isoP (pg/mg of protein)	0.00053 (0.00027–0.00099)	0.00052 (0.00014–0.0011)	ns
	8-OHdG (pg/mg of protein)	0.00074 (0.00028–0.0013)	0.00079 (0.00065–0.00088)	ns
	PC (nmol/mg of protein)	0.05 (0.035–0.07)	0.058 (0.02–0.08)	ns
	AOPP (μmol/mg of protein)	0.032 (0.028–0.045)	0.028 (0.016–0.042)	ns

*SMG, submandibular gland; PG, parotid gland; M, median; min, minimum; max, maximum; ns, not significant; HFD-IR, high fat diet insulin resistant rats; C, control group; 8-isoP, 8-isoprostanes; 8-OHdG, 8-D-hydroxyguanosine; PC, protein carbonyl groups; AOPP, advanced oxidation protein products*.

### The effect of high fat feeding on histological observation of salivary glands

**Figure 4:**
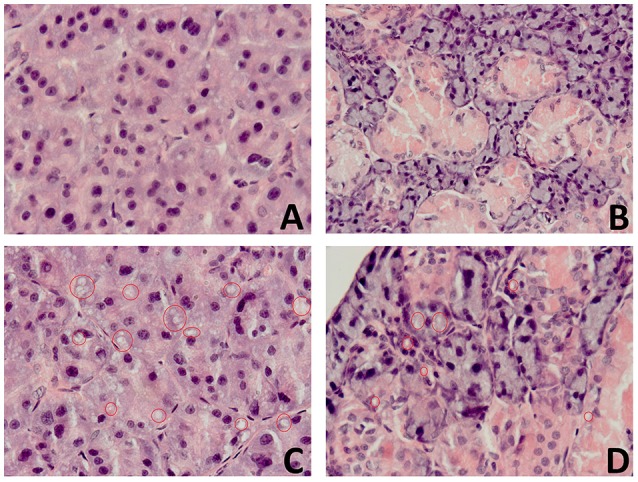
**The effect of high fat feeding on histological observation of salivary glands. (A)** —parotid gland of the control rats **(C)**, **(B)**—submandibular gland of the control rats **(C)**, **(C)**—parotid gland of the high fat diet insulin resistant rats (HFD-IR), **(D)**— submandibular gland of the high fat diet insulin resistant rats (HFD-IR); red circles indicate vacuoles.

In the high fat feeding group, acinar cells of both the submandibular and parotid glands demonstrated a degenerative changes in the form of vacuolation (Figure 4), wherein the significantly more vacuoles were observed in the parotid gland in comparison to the submandibular gland (Table 3).

**Table 3:** **The effect of high fat feeding on histological observation of salivary glands**.

	**Parotid glands *N* = 8**	**Submandibular glands *N* = 8**
C	8 (+)^*^	8 (+)^*^
HFD-IR	8 (++++)^◦^	7 (++), 1 (+++)

*+ single vacuoles in the cytoplasm of acinar cells*.

*++5–15% of the each section occupied by pathological changes*.

*+++ 16–30% of the each section occupied by pathological changes*.

*++++> 30% of the each section occupied by pathological changes*.

*C, control rats; HFD-IR, high fat diet insulin resistant rats*.

* *p < 0.05 control parotid/submandibular glands vs. HFD-IR parotid/submandibular glands*.

◦ *p < 0.05 HFD-IR parotid vs. HFD-IR submandibular glands*.

### Correlation

The Spearman correlation analysis showed that 4-HNE protein adduct (*p* = 0.001, *r* = 0.63) and PC (*p* = 0.03, *r* = 0.43) in parotid glands of HFD-IR rats were positively correlated with plasma insulin concentration. In parotid glands of HFD-IR rats positive correlations were also noted between plasma HOMA-IR and 4-HNE protein adduct (*p* = 0.02, *r* = 0.57) and 8-isoP (*p* = 0.003, *r* = 0.71). We also observed a positive correlation between plasma insulin concentration and 4-HNE protein adduct (*p* = 0.01, *r* = 0.48) as well as negative correlation between plasma FFA and protein concentration (*p* = 0.02, *r* = −0.43) in submandibular glands of HFD-IR rats.

## Discussion

Salivary glands are responsible for maintaining oral cavity homeostasis. Their dysfunction results in a change of the composition and amount of saliva provided to the oral cavity. If the pathology persists for a longer period of time, these changes may impair oral health. Convincing evidence has established the role of ROS in the pathogenesis and development of salivary glands pathology in diabetes, obesity and insulin resistance. It is well documented that diabetes mellitus, obesity and IR alter the antioxidants function of salivary glands (Nogueira et al., 2005; Deconte et al., 2011; Zalewska et al., 2013, 2014b, 2015; Narotzki et al., 2014). Oxidative damage to lipids, proteins and DNA included in salivary glands cellular organelles was reported both in experimental diabetes in animals (Deconte et al., 2011; Knaś et al., 2016a) and in diabetic (Al-Rawi, 2011) and obese patients (Narotzki et al., 2014; Knaś et al., 2016b). However, in conjunction with a limited number of the research articles, the phenomenon of OS in the salivary glands in the course of IR still has not been sufficiently explored.

Evidence showed that chronic high fat feeding induces obesity, IR (Ebertz et al., 2014; Matczuk et al., 2016), as well as increases general body OS and oxidative damage (Furukawa et al., 2004). It is not surprising that the HFD in our study resulted in a significant increase in the body weight of rats, a significant increase in plasma glucose, insulin, and fatty acids concentrations as well as all the examined parameters of oxidative stress. We have also shown significantly higher medians of the HOMA-IR index in the group of HFD-IR rats (*p* < 0.00001) as compared to rats fed a standard diet, which confirmed that chronic high fat feeding decreased whole body insulin sensitivity. Based on the available literature and guidelines on diagnosing insulin-resistance (Qu et al., 2008; Gan et al., 2013; Ebertz et al., 2014) our results confirmed IR in the group of rats fed a high fat diet.

The control of ROS stationary level is the result of a balance between their production and their elimination. Disequilibrium in this balance causes OS, which is usually accompanied by oxidative damage defined as “biomolecular damage caused-by attack of ROS upon the constituents of living organisms.” As was mentioned in the introduction, ROS can damage all classes of molecular cell components. Therefore, any single redox biomarker in isolation may be of limited value in diagnosis, staging, and prognosis of the oxidative stress-related human diseases. Many approaches for the assessment of oxidatively changed cellular components were selected. We used the most common assessment to evaluate oxidative damage: oxidized lipids (8-isoP and 4-HNE protein adduct), proteins (AOPP, PC), and DNA (8-OHdG).

Our study showed that both parotid and submandibular glands of HFD-IR rats had impaired ability to maintain normal redox balance compared to the data obtained in the salivary glands of the insulin-sensitive control rats, resulting in a significant increase in the level of oxidized biomolecules in comparison to the salivary glands from control group.

However, a greater percentage increase in the concentration of lipid oxidation products (parotid gland (4-HNE protein adduct ↑39%, 8-isoP ↑27%) vs. submandibular gland (4-HNE protein adduct ↑15%, 8-isoP ↑20%), as well as new types of oxidatively modified components (AOPP ↑25, PC ↑32, and 8-OHdG ↑18%) in the parotid glands of HFD-IR rats as compared to the control proved that parotid glands are subjected to more intense OS, or they are more susceptible than submandibular glands to oxidant attack generated in the course of HFD induced IR. In addition, selective, from all oxidation products, a significant increase in lipoperoxidation products levels in the submandibular glands of rats feed with HFD vs. control prove once again that these glands are subject to lower intensity OS than parotid glands. It has been shown that the earliest sign of developing OS are the oxidative lipid modification products, due to the fact that the cell membrane and its lipids are the first to be exposed to the harmful effects of the free radicals. As the concentration of ROS further increases, the concentration of lipid peroxidation products grow and also proteins undergo oxidation and later DNA (Ayala et al., 2014).

The observed a significant increase in 8-OHdG level in the parotid glands of high fat feeding insulin resistant rats may have serious consequences for oral and also general health. The formation of 8-OHdG is the best known DNA damage occurring via OS and it is considered a “bio indicator” of carcinogenesis (Birben et al., 2012). It was shown that IR is a risk factor for the development of not only cardiovascular complications but also salivary glands tumors (Suba et al., 2005). However, the authors did not specify which salivary glands are more pronounced but a claim that a better control of IR seems to be necessary not only to reduce the cardiovascular risk but also prevent tumor promotion.

The present experiment does not explain the causes of a different course of OS in both salivary glands, however, it may be partly related to the observations of Zalewska et al. (2014b). Their report revealed that the antioxidant system of the parotid glands of rats feed with a HFD is more deficient to combine ROS as compared to submandibular one (Zalewska et al., 2014b). On the other hand, more intense oxidative damage to the parotid glands of HFD-induced insulin resistance rats may also be related to their physiological morphology, namely the presence of adipocytes in the parenchyma of the parotid glands (Amano et al., 2012). It not without significance is also the fact that only parotid glands displayed weight alteration from chronic high fat feeding, which was also observed in STZ diabetes (Mori et al., 1990) and obese (Bozzato et al., 2008) as well as type 2 diabetic (Carda et al., 2006) patients. The present study did not explain the nature of severe intracytoplasmic vacuolization in the parotid glands (with minimal changes in submandibular glands); however these vacuoles appeared to be a lipid nature since they were removed during fixation and processing of the samples. It was also shown that obese and type 2 diabetic parotid glands exhibited a significant enlargement, which is a result of enhanced storage of lipid droplets/adipocytes in the parotid parenchyma. Exposure to HFD leads to activation of inflammatory signaling in adipocytes and macrophages (↑ secretion of pro-inflammatory cytokines (TNFα, IL-6, IL-1β), activation of NADPH oxidase, and ↑ ROS production). Moreover, high fat feeding causes adipocyte to release monocyte chemoattractant protein-1 (MCP-1) that attracts monocytes in adipose tissue and transforms them in tissue resident inflammatory M1 phenotype macrophage (Solinas and Karin, 2010). The deepening inflammation, resulting in a further increase in production of the free radicals, which in the weakening of the antioxidant barrier leads to the oxidative damage to the organ and its dysfunction.

There was no obvious difference in the secretory response of the salivary glands under no stimulation, phenomena observed in obese patients (Knaś et al., 2016b). On the other hand, pilocarpine-stimulated whole saliva flow rate was 45% decreased in HFD-IR rats, compared to the control group. These results could suggest that salivary glands of HFD-IR rats have lowered susceptibility to muscarinic receptor stimulation, as was presented in STZ rats (Watanabe et al., 2001). On the other hand, if we assume that after stimulation only parotid glands increase their secretion (Zalewska et al., 2014a), reduced secretion of the stimulated saliva may be a result of severe acinar cell vacuolation in the parotid glands, and thus leads to reduction in the active secretory surface of the salivary glands. Affected stimulated saliva flow may be also due to the existence of disturbances at the level of neurotransmission. It can be assumed that extracellular matrix reconstruction caused by the parenchyma vacuolation of the salivary glands, chronic low grade inflammation (observed in parotid glands of obese and type 2 diabetes mellitus), ROS and acinar degeneration prevent function and/or communication of residual of the neural and the residual secretory units; however, to confirm or exclude this mechanism requires further studies.

In contrast to the saliva secretion, the mechanism involved in the synthesis/secretion of protein seems to be more sensitive to the effects induced by a high-fat diet. Protein concentration was significantly depressed in both glands, wherein in parotid glands was more disrupted than in submandibular one. There may be many reasons for reduced synthesis, secretion and total protein concentration. One of them is the reduced tissue sensitivity to insulin, which can be confirmed by a negative correlation between the plasma FFA level and the concentration of protein in the submandibular glands. It has been demonstrated that an increased plasma FFA level seriously affects insulin signaling pathway and contributes to the development of intracellular insulin resistance. Not without significance can be a morphologic changes as well as described above disturbances of neurotransmission.

Noteworthy is the absence of any correlation between the parameters of oxidative damage to both salivary glands and parameters of oxidative damage recorded in the serum. This may prove that oxidatively modified cellular components arise directly in the salivary glands, and they are not a result of diffusion of oxidation products from the blood vessels. However, we have shown a positive correlation between serum levels of insulin and 4-HNE protein adduct and PC in the parotid glands as well as concentrations of insulin and 4-HNE protein adduct in submandibular glands, which is in agreement with the data that insulin deficiency via enhancement of fatty acyl coenzyme A oxidase results in an increase in the oxidative environment and the same oxidative damage (Schönfeld et al., 2009). We also observed the positive correlation between 4-HNE protein adduct and 8-isoP in parotid glands of HFD-IR rats and plasma HOMA-IR, which may suggest that only parotid lipid peroxidation products concentration increase as a function of insulin resistance, whereas parotid protein carbonyls, 8-OHdG and submandibular lipid peroxidation products are independent of insulin sensitivity.

They are a few limitation to our study. Most importantly, salivary glands: oxidative damage, dysfunction and morphological changes could be a consequences of high fat feeding or insulin resistance *per se* as well. Only kinetic studies could address this question. Secondly, they are a lot of other markers of ROS induced modification, and OS markers used by us are only the most frequently used. Using other markers of OS may partially or completely change our observations and conclusions. The applied animal model, very helpful in explaining occurring pathology, cannot be postponed directly to the humans.

## Conclusion

In this study we have shown that high fat feeding results in the salivary gland's OS and oxidative damage as well as in the salivary glands dysfunction. We observed that mechanisms involved in the synthesis/secretion of proteins are affected in the parotid and submandibular glands of IR rats, however only stimulated secretory ability in the salivary glands is depressed in the course of HFD-induced insulin resistance. Moreover, we noted that the oxidative/antioxidative balance in both glands of IR rats shifted toward the oxidative status; however the parotid glands are exposed to a greater intensity oxidative stress. We also demonstrated that insulin resistance results in oxidative damage to DNA, proteins, and lipids, however oxidative damage in the salivary glands arises independently from the general OS as well as only the parotid lipid peroxidation products concentration seems to increase as a function of insulin resistance. IR is a prediabetic state, so one can see that salivary glands dysfunction manifests early in diabetes progression and it is detectable in prediabetic state. So IR should be taken seriously by the dentists, because at this stage diabetes can adversely affect the health of the oral cavity. Summarizing, OS may be a major phenotypic hallmark in the salivary insulin resistance. We believe that antioxidants supplementation could alleviate and/or prevent the damaging effects of OS and oxidative damage in patients with insulin resistance, improving function of the salivary glands as well as homeostasis of the oral cavity.

## Author contributions

UK conceptualized, interpreted of data, wrote of the manuscript. MM conceptualized, did laboratory determination, did performance of the graphic part of the manuscript. AM did histological examination and interpreted these data. JM conceptualized, interpreted of data. MK- did statisctical analysis. PŻ conceptualized, did literature survey. MŻ and JB did literature survey, final approval of the version to be published. AZ conceptualized, did laboratory determination, interpreted of data, wrote of the manuscript.

### Conflict of interest statement

The authors declare that the research was conducted in the absence of any commercial or financial relationships that could be construed as a potential conflict of interest.
